# Research on the characteristics and influencing factors of the spatial correlation network of cultivated land utilization ecological efficiency in the upper reaches of the Yangtze River, China

**DOI:** 10.1371/journal.pone.0297933

**Published:** 2024-02-26

**Authors:** Wei He, FeiFan Wang, Ni Feng

**Affiliations:** 1 Key Lab of Land Resources Evaluation and Monitoring in Southwest, Ministry of Education, Sichuan Normal University, Chengdu, China; 2 College of Geography and Resources Science, Sichuan Normal University, Chengdu, China; Shenzhen University, CHINA

## Abstract

Researching the structural characteristics of the spatial correlation network of cultivated land utilization ecological efficiency is of great significance to China’s food security and agricultural green and low-carbon development. Taking 47 cities (autonomous prefectures) in the upper reaches of the Yangtze River as the research object, the ecological efficiency of cultivated land utilization from 2010 to 2020 was measured based on the unexpected output model (Super SBM), and the spatial correlation matrix was constructed using the revised gravity model. The structural characteristics of the spatial correlation network were analyzed using the social network model (SNA), and finally, the factors affecting the spatial correlation network of cultivated land utilization ecological efficiency in the upper reaches of the Yangtze River were analyzed through the quadratic assignment procedure (QAP) model. The results show that: (1) the ecological efficiency of cultivated land utilization in the upper reaches of the Yangtze River has been increasing year by year, but the overall level is low, and there is a large gap among provinces. Sichuan Province has the highest average value of 0.605, and Yunnan Province has the lowest average value of 0.359. (2) The ecological efficiency of cultivated land utilization in the upper reaches of the Yangtze River has broken through the provincial boundaries and has formed an obvious spatial correlation network, but the overall density is low, and the network is still relatively loose, needing further development and improvement. Chengdu, Yibin, Luzhou, and other cities are located in the center of the network and have formed four cohesive subgroups. (3)The differences in the level of agricultural economic development, the rural per capita disposable income, the differences in agricultural mechanization intensity, the regional population differences, and spatial adjacency have an impact on the spatial network of ecological efficiency of cultivated land utilization in the upper reaches of the Yangtze River. The difference in the level of agricultural economic development, the rural per capita disposable income, and the differences in agricultural mechanization intensity are negatively correlated, while the regional population differences are positively correlated with spatial adjacency.

## Introduction

The spatial correlation of cultivated land utilization ecological efficiency refers to the exchange of cultivated land ecological efficiency in different regions through direct or indirect forms and the resulting spatial interaction between regions [1–3]. Under the joint promotion of the current agricultural production marketization, scale, and cross-regional coordinated development strategies, various agricultural production factors are accelerating in space and spreading, and the ecological efficiency of cultivated land utilization has begun to show obvious spatial effects [4–7], and have begun to form a complex spatial network structure. Scientific measurement of the Ecological efficiency of cultivated land utilization and comprehensive examination of its spatial association network characteristics and driving factors are of great significance for improving the Ecological efficiency of cultivated land utilization and promoting the development of green agriculture.

Eco-efficiency (Eco-efficiency) was first proposed by German scholars Schaltegger and Sturm in the 1990s [8]. Given the proposal of China’s sustainable agricultural development strategy, research on the ecological efficiency of cultivated utilization has received increasing attention from scholars. At present, the ecological efficiency of cultivated land utilization is defined as "The extent to which economic and social benefits can be maximized and environmental pollution minimized with a certain input of production factors in the process of cultivated land utilization, and its pursuit of "resource—economy—environment " [9, 10]. In the measurement of cultivated land ecological efficiency, commonly used methods include stochastic frontier analysis [11], life cycle method [12], ecological footprint method [13], principal component analysis [14], and Data envelopment analysis (DEA) method [15]. The literature mostly uses the SBM model, including unexpected output, to measure the ecological efficiency of cultivated land utilization [16, 17]. In terms of influencing factor analysis, current studies mainly use the Tobit model [18, 19], geographic detectors [20, 21], spatial econometric models [22, 23], and other methods to analyze the effects of rural per capita disposable income, urbanization rate and other factors on the ecological efficiency of cultivated land utilization and their spatial heterogeneity. With the continuous advancement of regional coordinated development, an increasing number of studies have been conducted to explore the spatial effects of cultivated land utilization ecological efficiency from the perspective of time and space. Scholars such as Wu Haoyu found that China’s interprovincial cultivated land utilization efficiency was positively correlated in space [24], and its spatial correlation pattern experienced a process of "multicore distribution "-" one belt and one area "-" low pole dominance "; Yan Mingtao and other scholars found that farmer income level, per labor sown area and planting structure, have a positive and direct effect on agricultural ecological efficiency in the region, the urbanization rate and agricultural machinery density has a negative direct effect on the agricultural ecological efficiency of the region [25].

Current studies have confirmed that there is a spatial spillover relationship in the ecological efficiency of cultivated land utilization, but they have not completely explained the spatial correlation model, characteristics, and influencing factors of the ecological efficiency of cultivated land utilization, the research on the ecological efficiency of cultivated land utilization based on the network perspective are still insufficient. Social Network Analysis (SNA) is one of the methods for studying spatial relationships from the perspective of social networks, and it is based on flow data and analyzes the spatial correlation between nodes through centrality analysis and cohesive subgroups. It has been widely used in sociology [26, 27], economics [28, 29], management, resources and the environment [30] and other fields. Using the social network analysis method to carry out research on the Ecological efficiency of cultivated land utilization can more objectively reflect the spatial correlation model and characteristics of the Ecological efficiency of cultivated land, reveal its spatial effect and impact, and enrich the research on Ecological efficiency of cultivated land utilization.

The Yangtze River Economic Belt is an important economic corridor and ecological treasure house in China. The development strategy of the Yangtze River Economic Belt is an important measure to promote the development of China’s agricultural economy. Agriculture is the basic industry of the development strategy of the Yangtze River Economic Belt. As an important method of production, cultivated land promotes the growth of cultivated land. Green and low-carbon utilization is of great significance to the green and high-quality development of agriculture in the Yangtze River Economic Belt [31, 32]. This paper takes 47 prefectures (autonomous prefectures) in Sichuan, Chongqing, Guizhou, and Yunnan provinces in the upper reaches of the Yangtze River in 2010 and 2020 as the research object and uses the super efficiency SBM model to measure the ecological efficiency of cultivated land utilization and the revised gravity model. The gravitational matrix of the ecological efficiency of cultivated land utilization is constructed, the structural characteristics of the spatial correlation network of the ecological efficiency of cultivated land utilization are analyzed by using the social network analysis method, and the driving factors are explored through the QAP model, aiming to provide reference and support for the coordinated development of green low-carbon agriculture and regional agriculture in the upper reaches of the Yangtze River and provide a theoretical basis for the high-quality utilization of cultivated land.

## Overview of the study area and data sources

### Overview of the study area

**Fig 1:**
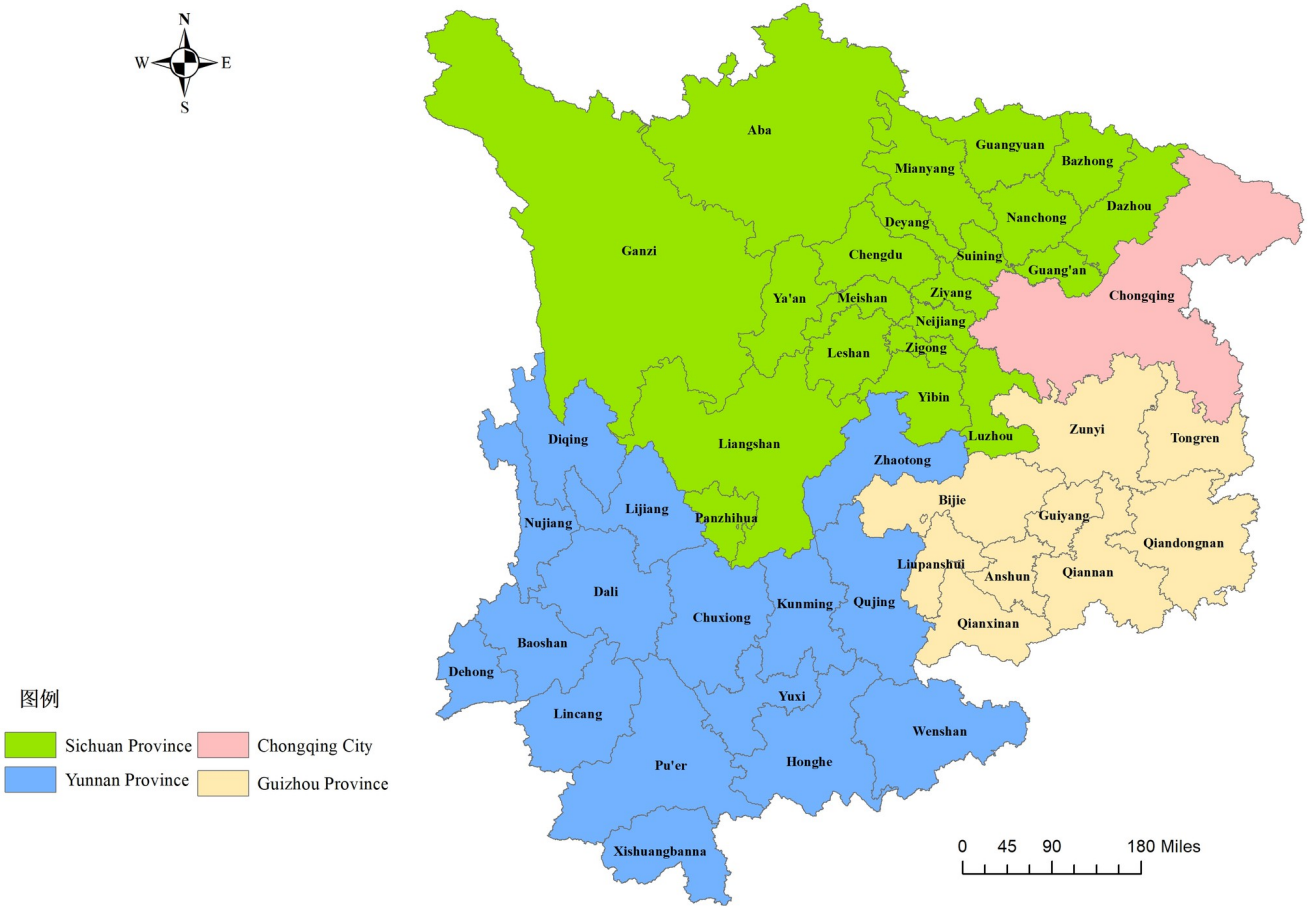
Location map of the upper reaches of the Yangtze River. The map was prepared with ArcGIS10.1 (https://resources.arcgis.com/en/help/). The administrative boundary was republished from [34] under a CC BY license, with permission from Resource and Environment Science and Data Center (https://www.resdc.cn/), original copyright 2023.

The upper reaches of the Yangtze River involve Chongqing, Sichuan, Tibet, Qinghai, Yunnan, Guizhou, and other places. Most of the hydropower resources in the Yangtze River Basin are concentrated in this region. Considering the data availability, this paper takes Sichuan, Yunnan, Guizhou, and Chongqing as the main research areas (Fig 1). The upper reaches of the Changjiang River have a subtropical monsoon climate, it is rich in water resources, crisscrossing canyons, and mostly mountains and hills. Among these areas, the mountainous areas of Guizhou account for 92.5% of the total area of the province, 94% of Yunnan, and 96.26% of Chongqing Sichuan, accounting for 97.46%. Due to the constraints of terrain and economic development, the efficiency of cultivated land utilization in the upper reaches of the Yangtze River has always been low. Therefore, it is of great significance to study the ecological efficiency of the cultivated land utilization in the upper reaches of the Yangtze River and explore ways to improve it to ensure national food security.

### Data sources

The basic data of this paper mainly come from the "Sichuan Provincial Statistical Yearbook" (2011–2021), "The Yunnan Provincial Statistical Yearbook" (2011–2021), "The Chongqing Statistical Yearbook" (2011–2021), and "Guizhou Statistical Yearbook" (2011–2021) 2021), as well as yearbooks and statistical bulletins of various cities (autonomous prefectures). The distance between cities (autonomous prefectures) is calculated using the geographic coordinates obtained by the distance function in ArcGIS software.

## Research methods

### Calculation method of the ecological efficiency of cultivated land utilization

#### Input-output index system of cultivated land utilization ecological efficiency

According to the definition of the ecological efficiency of cultivated land utilization, the utilization of cultivated land should not only ensure economic and social benefits but also reduce environmental pollution and waste of resources. Referring to previous research results [1, 7] and the availability of data on Prefecture-level cities (autonomous prefectures) in the upper reaches of the Yangtze River, an input-output indicator system of Ecological efficiency of cultivated land utilization in the upper reaches of the Yangtze River is constructed, as shown in Table 1. The input indicators include land input, labor input, agricultural machinery input, and chemical fertilizer input; the expected output indicators include the total grain output and the total agricultural output value; since the main pollution in grain production activities is carbon emissions, carbon emissions are taken as the undesired output. Carbon emissions are obtained by obtaining the carbon emission coefficient of the main carbon source of cultivated land utilization and the summation of the product of the total amount of various carbon sources [31].

**Table 1:** Input-output indicators of cultivated land Utilization ecological efficiency.

index	Indicator category	Indicator Interpretation
put in	land input	Sown area of crops (thousand hectares)
	labor input	Number of employees in the primary industry (10,000 people)
	Agricultural Machinery Input	Total power of agricultural machinery (10,000 kilowatts)
	Fertilizer input	Fertilizer application amount (10,000 tons)
output	expected output	Grain output (10,000 tons)
		Gross agricultural output value (100 million yuan)
	undesired output	Carbon emissions (10,000 tons)

#### Super SBM model considering the undesired output

Regarding the measurement and calculation of ecological efficiency, the current research mostly uses the model method for measurement and calculation. Data Envelope Analysis (DEA) is a parameter frontier method, but the traditional DEA model has the disadvantage of radial angle, and this will cause invalid slack variables and cannot be accurate. To measure the efficiency value when including unexpected output, the Super SBM model is based on the unexpected output from the perspective of non-direction and non-angle based on previous research [33], overcoming the problem of slack variables, incorporating the unavoidable undesired output problem in the process of cultivated land utilization into the model, and comprehensively measuring the relationship between input and output (including expected output and undesired output).

### Analysis method of spatial association network structure

#### Modified gravity model

The current social network analysis method mainly includes the Granger causality test and gravity model for determining the strength of spatial connections, but the Granger causality test is sensitive to the lag order and cannot describe the dynamic characteristics of the association network. Therefore, this paper uses the revised gravitational model to measure the gravitational value of the ecological efficiency of cultivated land utilization between cities [35]. The calculation formula is:

$$S_{ij}=\;K_{ij}\frac{M_{i}*M_{j}}{{D_{ij}}^{b}}$$ (1)

In the formula, **S**_**ij**_ indicates the connection strength between City i and city j; **M**_**i**_ and **M**_**j**_ represent the ecological efficiency of cultivated land utilization in city i and city j respectively; **K**_**ij**_ is the contribution rate, $K_{ij}=\frac{{\text{M}}_{\text{i}}}{{\text{M}}_{\text{i}}\text{+}{\text{M}}_{\text{j}}}$; **D**_**ij**_ indicates the geographical distance between cities i and j; and b is the distance attenuation coefficient used in the study of the relationship between regions, which usually takes a value of 2.

According to the current research, this paper sets the average value of the matrix as a threshold. If the connection strength is greater than this threshold, it is recorded as 1, indicating that there is a correlation. In contrast, if it is less than this threshold, it is recorded as 0, indicating that there is no spatial correlation, and finally, a 47 * 47 binarization matrix is formed.

#### Social network analysis

The social network analysis method is based on the relationship data between the research objects, and it is a scientific method to analyze the relationship network. The network analysis method can be used to discuss the structure and attribute characteristics of the network [36], including individual attributes and overall attributes.

This paper uses the three indicators of network density, network centrality, and cohesive subgroups in the social network analysis method to analyze the spatial correlation network structure characteristics of the ecological efficiency of cultivated land utilization in the upper reaches of the Yangtze River. Social network density reflects the sparsity of the spatial network. Social network centrality reflects the importance of each node in the spatial network, including three indicators such as point-degree centrality, betweenness centrality, and Proximity to centrality. Cohesive subgroup analysis to identify potential network groups.

*Social network density*. Social network density is used to measure the closeness of each node in the network. If the network density is higher, it means that the 47 cities (autonomous prefectures) in the upper reaches of the Yangtze River are more closely connected. The impact is also stronger. The calculation formula is:

$$\text{D}=\frac{\text{m}}{\text{n}\;(\text{n}-1)}$$ (2)

where D represents the overall network density of cultivated land utilization eco-efficiency in the upper reaches of the Yangtze River; m is the number of relationships or connections that exist in the network; and n is the number of nodes (number of cities) in the network.

*Social network centrality*.

1. Point-degree centrality
  Point-degree centrality is used to measure the radiation or agglomeration ability of nodes in the network. The higher the point-degree centrality, the closer the node is to the center of the network, and the stronger the effect. Its calculation formula is:
$$\text{C}(n_{i})\;=\frac{\sum_{j=1}X_{ij}}{\left(d-1\right)}$$ (3)
  In the formula, C (***n***_***i***_) represents the point-degree centrality of network node i, which is the connection strength index between node i and node j, and d is the number of nodes in the network.
2. Betweenness centrality
  The betweenness centrality indicates the betweenness of a certain node. The higher the betweenness centrality is, the stronger its regulating effect and the calculation formula is:
$$C_{N}\left(n_{i}\right)\;=\frac{2\sum_{j=1}^{N}\sum_{k=1}^{N}b_{jk}\left(i\right)}{N^{2}-3N+2}$$ (4)
  In the formula, j ≠ k ≠ i, j < k, and is the number of shortest paths from node j to node k in the network that passes through node i. ***b***_***jk***_ is the number of shortest paths from node j to node k, and d is the number of nodes in the network.
3. Proximity to centrality
  Proximity centrality mainly reflects the ability of a node in the spatial correlation network to not be “controlled” by other nodes. The higher the proximity centrality, the closer the distance between this node and other nodes in the spatial correlation network of cultivated land utilization ecological efficiency. Recently, it has been shown to transmit information more conveniently in the network and promote the flow of agricultural elements. The calculation formula is:
$$C_{A}\left(n_{i}\right)\;=\frac{\sum_{j=1}^{N}d_{ij}}{N-1}$$ (5)
  In the formula, ***C***_***A***_ (***n***_***i***_) represents the proximity centrality and represents the convenient distance between node i and node j.

*Cohesive subgroup analysis*. Social network cohesion subgroups can reflect the relational position of different nodes in the network, revealing the spatial structure of the network. This method can depict and reveal the state of subgroups within the network [37]. By dividing the network into subgroups, it is possible to understand the spatial organization and architecture of the ecological efficiency network of cultivated land utilization in the upper reaches of the Yangtze River.

#### Quadratic assignment procedure (QAP) model

Since the variables involved in social network analysis are all relational data, the correlation between nodes constitutes a binary relational matrix. Relational data expressed in the form of a matrix usually have problems such as autocorrelation and multicollinearity. Therefore, the traditional measurement method will cause large variance and standard deviation of parameter estimates, while the QAP model is a nonparametric replacement test method for relational data. The main steps are divided into two steps: one is to investigate the correlation between the independent variables, and the other is to investigate the regression relationship between the dependent variables and the independent variables [38]. The calculation formula is:

$$R=\left(X1,X2,\ldots ,Xn,S\right)$$ (6)

In the formula, *R* is the spatial correlation matrix of ecological efficiency of cultivated land utilization in the upper reaches of the Yangtze River, *X1*, *X2*, and *Xn* are the influencing factors, and *S* is the spatial adjacency.

## Results and analysis

### Ecological efficiency of cultivated land utilization analysis

This paper uses Maxdea 8 ultra software, based on the input-output index system of cultivated land utilization ecological efficiency, using the unexpected output SBM model to analyze data from 2010–2020 and calculate the ecological efficiency of cultivated land utilization in 47 cities (autonomous prefectures) in the upper reaches of the Yangtze River in 2010-2020(Table 2).

**Table 2:** The average value of ecological efficiency of cultivated land utilization in the upper reaches of the Yangtze River in 2010 and 2020.

years	Sichuan Province	Chongqing	Guizhou Province	Yunnan Province	average
2010	0.530	0.424	0.311	0.319	0.396
2011	0.552	0.430	0.236	0.300	0.380
2012	0.519	0.444	0.275	0.313	0.388
2013	0.526	0.452	0.281	0.327	0.397
2014	0.517	0.451	0.337	0.333	0.410
2015	0.565	0.455	0.373	0.366	0.440
2016	0.611	0.486	0.400	0.371	0.467
2017	0.614	0.493	0.433	0.378	0.479
2018	0.650	0.518	0.465	0.373	0.501
2019	0.728	0.542	0.508	0.416	0.549
2020	0.844	0.589	0.603	0.454	0.623
Average	0.605	0.481	0.384	0.359	0.457

**Fig 2:**
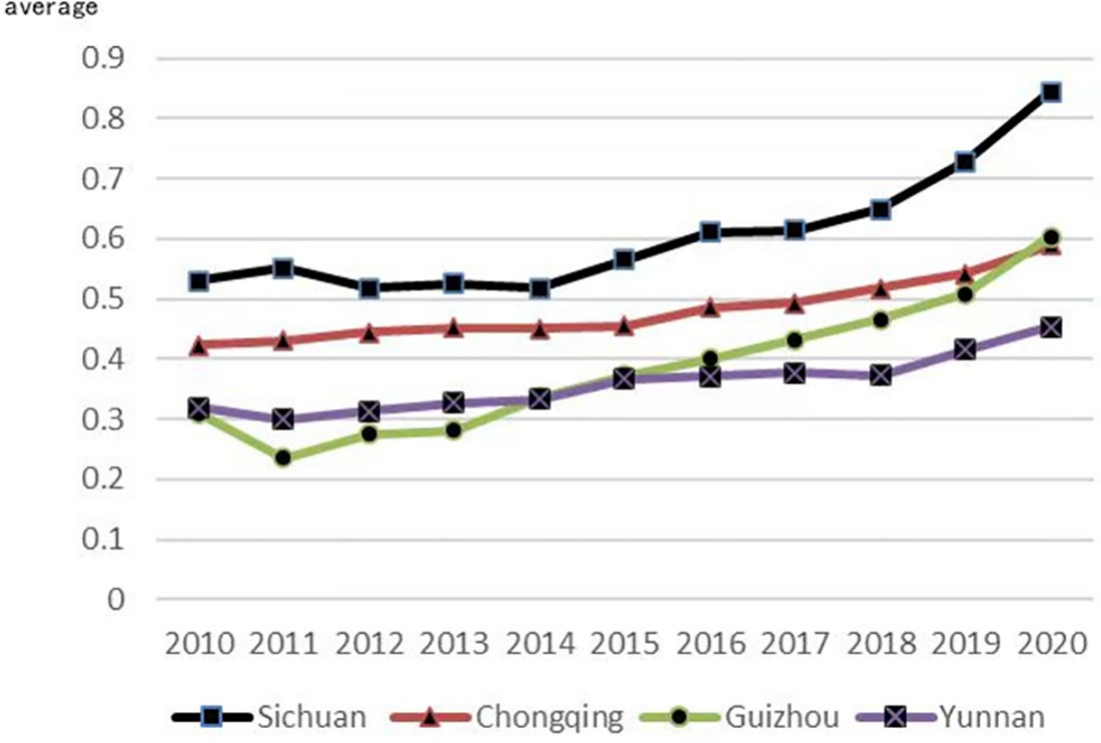
Changing trend of ecological efficiency of cultivated land utilization in the upper reaches of the Yangtze River from 2010 to 2020.

According to the previous findings, this paper divides the ecological comprehensive efficiency of cultivated land utilization into five grades by using the natural breakpoint method: high efficiency (P≥1), relatively high efficiency (0.8≤P<1), medium efficiency (0.6 ≤P<0.8), low efficiency (0.4≤P<0.6), and low efficiency (P<0.4). Table 2 shows that the ecological efficiency of cultivated land utilization in the upper reaches of the Yangtze River in 2010 and 2020 was generally low, with an average value of 0.457, indicating that the ecological efficiency of cultivated land utilization in the upper reaches of the Yangtze River was generally at a low level. From the perspective of time dimension evolution, the ecological efficiency of cultivated land utilization in the upper reaches of the Yangtze River has shown a gradual upward trend, rising from 0.396 in 2010 to 0.623 in 2020, and gradually rising from low efficiency to medium efficiency, indicating that there is still a large development space for resource-intensive and environmental protection. From four provinces in 2010, looking at the average ecological efficiency of cultivated land utilization in 2020, Sichuan Province has the highest average value of 0.605, followed by Chongqing at 0.481, Guizhou Province at 0.384, and Yunnan Province the lowest at 0.359. Among them, only the average value of Sichuan Province has reached medium efficiency, while the average value of Guizhou Province and Yunnan Province is low efficiency. The changing trend is shown in Fig 2. The average value of the ecological efficiency of cultivated land utilization in the four provinces in the upper reaches of the Yangtze River has been increasing year by year, and Sichuan Province had the largest change in 2010. The increase was relatively slow in 2017 and then it significantly accelerated, rising to 0.844 in 2020, reaching a relatively high efficiency; Chongqing City has steadily increased year by year, and the average ecological efficiency of cultivated land utilization has risen from 0.424 in 2010 to 0.589 in 2020 but has always been in the lower efficiency range; the average value of the ecological efficiency of cultivated land utilization in Yunnan Province has not changed much, and the cities have all been in the low-efficiency range; the ecological efficiency of cultivated land utilization in Guizhou Province has been increasing steadily, from 0.311 in 2010 to 0.603 in 2020, from low efficiency to medium efficiency.

**Fig 3:**
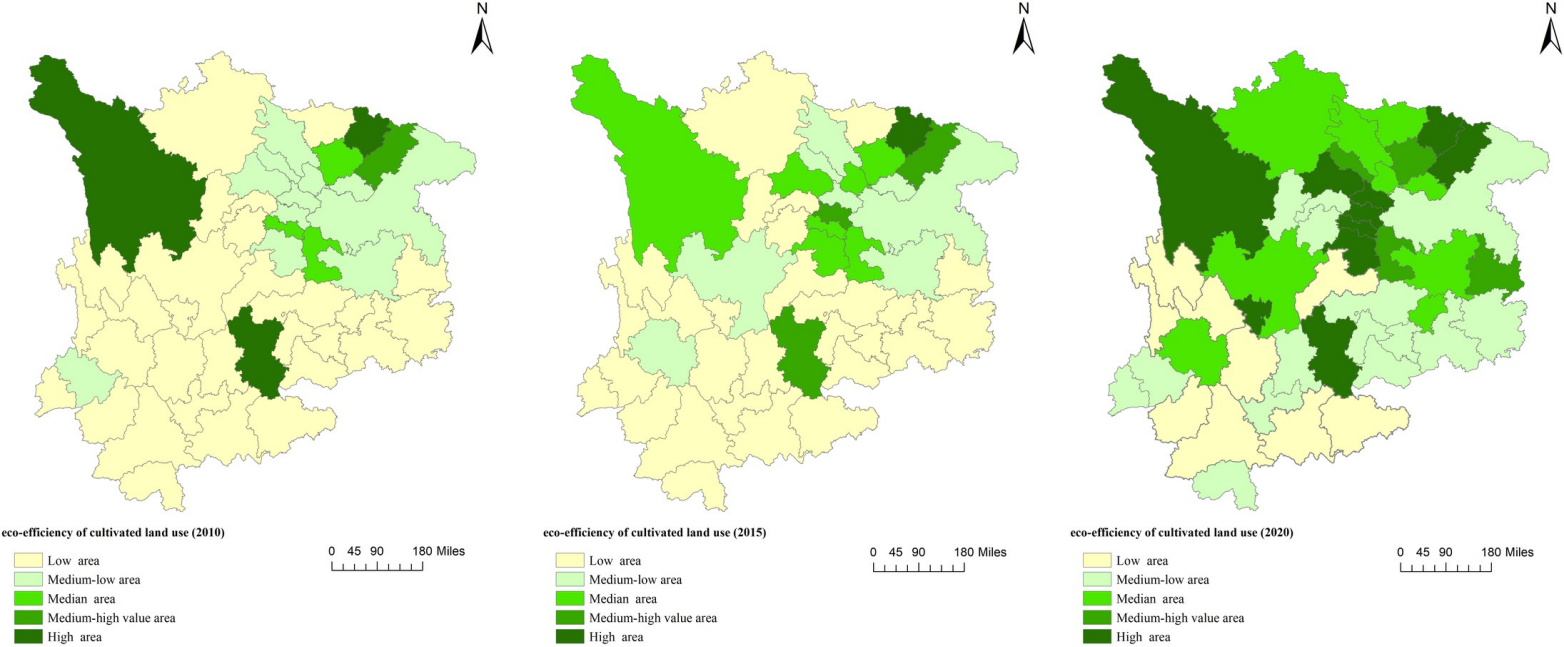
Spatial distribution of cultivated land utilization ecological efficiency in the upper reaches of the Yangtze River. The map was prepared with ArcGIS10.1 (https://resources.arcgis.com/en/help/). The administrative boundary was republished from[34] under a CC BY license, with permission from Resource and Environment Science and Data Center (https://www.resdc.cn/), original copyright 2023.

By further exploring the trend of internal differences in the ecological efficiency of cultivated land utilization in the upper reaches of the Yangtze River, this paper draws the spatial distribution of the ecological efficiency of cultivated land utilization in 2010, 2015, and 2020 in 47 cities (autonomous prefectures) in the upper reaches of the Yangtze River using ArcMap 10.8 software. As shown in Fig 3, the ecological efficiency of cultivated land utilization in the upper reaches of the Yangtze River shows obvious spatial differentiation characteristics.

In 2010, the overall ecological efficiency of cultivated land utilization in the upper reaches of the Yangtze River was generally not high, with high- and relatively high-efficiency areas accounting for only 8.5% of the total number and low-efficiency areas accounting for 61.7%. Among them, there were only three high-efficiency areas, namely, Ganzi Prefecture, Bazhong City, and Qujing City; there was only one high-efficiency area, which is Dazhou City; there were only three medium-efficiency areas, namely, Luzhou City, Nanchong City, and Zigong City, and they are all located in Sichuan Province; it is mainly concentrated on the northeast of the study area, mostly in the Sichuan-Chongqing region; in Yunnan Province, except for Qujing City, which has achieved high efficiency, the rest of the region is a low-efficiency area; in Guizhou Province, except for Zunyi City, which is a low-efficiency area, the rest of the area is the low-efficiency district.

In 2015, the overall ecological efficiency of cultivated land utilization in the upper reaches of the Yangtze River did not change much, and only Bazhong City reached a high level of efficiency. The number of areas has increased significantly, but most areas in Yunnan and Guizhou provinces are still at a low level of efficiency, indicating that the cultivated land in Yunnan and Guizhou still has great potential in terms of economic benefits.

In 2020, the overall ecological efficiency of cultivated land utilization in the upper reaches of the Yangtze River will be further improved, and the number of high- and relatively high-efficiency areas will increase significantly, accounting for 29.8% of the total, mostly distributed in Sichuan Province. The efficiency has reached the medium efficiency level and above. At the same time, the number of low-efficiency areas in the study area has decreased significantly, accounting for only 19% of the total, and they are all located in Yunnan Province, while Sichuan Province and Guizhou Province have no low-efficiency areas.

### Analysis of the characteristics of the spatial correlation network of cultivated land utilization ecological efficiency

Through social network analysis, the spatial correlation between nodes can be demonstrated. This paper explores the spatial network characteristics of cultivated land utilization eco-efficiency in the upper reaches of the Yangtze River based on three aspects: network density analysis, network centrality analysis, and cohesive subgroup analysis.

#### Network density analysis

**Fig 4:**
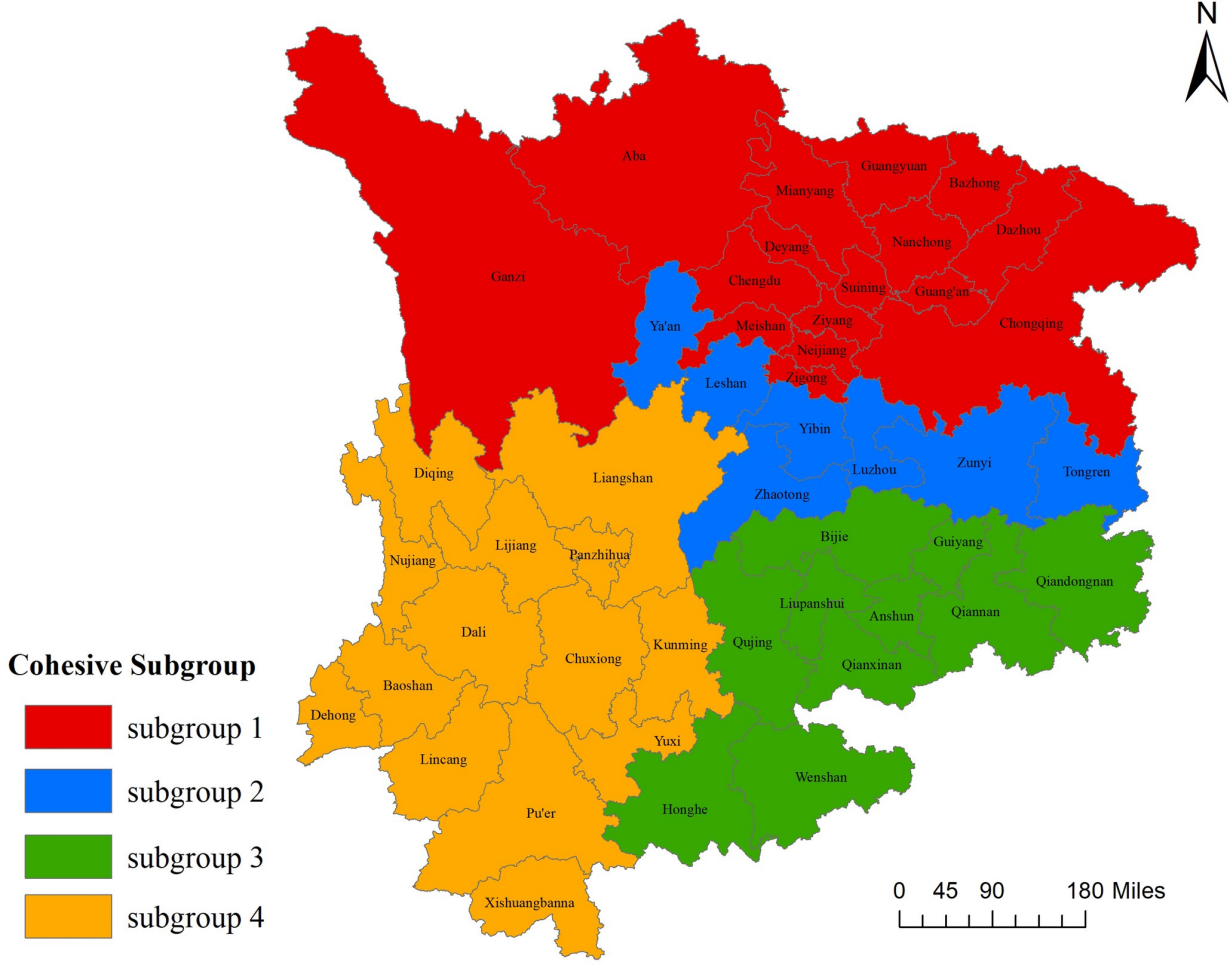
Evolution of the spatial correlation network of cultivated land utilization ecological efficiency in the upper reaches of the Yangtze River from 2010 to 2020. The map was prepared with ArcGIS10.1 (https://resources.arcgis.com/en/help/). The administrative boundary was republished from[34] under a CC BY license, with permission from Resource and Environment Science and Data Center (https://www.resdc.cn/), original copyright 2023.

Revised gravity model, this paper constructs the spatial correlation matrix of cultivated land utilization ecological efficiency in the upper reaches of the Yangtze River and uses ArcGIS software to draw the topological structure of the spatial network in 2010, 2015, and 2020. As shown in Fig 4, the ecological efficiency of cultivated land utilization in the upper reaches of the Yangtze River has broken through the traditional spatial and geographical proximity spillover attributes, showing a certain spatial network structure. In this paper, the overall network density of the upper reaches of the Yangtze River in 2010, 2015, and 2020 measured by Gephi software is 0.183, 0.184, and 0.191, respectively, indicating that the overall spatial correlation of the ecological efficiency spatial network of cultivated land utilization in the upper reaches of the Yangtze River is gradually strengthened. However, the spatial correlation network density of cultivated land utilization ecological efficiency in the upper reaches of the Yangtze River is at an overall low level, the growth rate is slow, and the spatial correlation network of cultivated land utilization ecological efficiency is still relatively loose, needing further development and improvement. At the same time, the status of each city in the network has obvious spatial heterogeneity. It can be seen that cities located in the northeast of the study area, such as Yibin, Zigong, Suining, and Chengdu, are at the center of the network. The number and intensity of spatial correlation relationships are significantly better than those in Yunnan and Guizhou, indicating that the exchange of agricultural production factors in Sichuan Province is more closely connected than in other regions.

#### Network centrality analysis

Centrality indicators in social network analysis mainly include degree centrality, proximity centrality, and betweenness centrality. This paper uses Ucinet 6 software to calculate the centrality indicators of the ecological efficiency of cultivated land utilization in cities (autonomous prefectures) in the upper reaches of the Yangtze River in 2020, and the results are listed in Table 3.

**Table 3:** Ranking of spatial network centrality of cultivated land utilization ecological efficiency in the upper reaches of the Yangtze River in 2020.

ranking	degree centrality	Proximity to centrality	betweenness centrality	ranking	degree centrality	Proximity to centrality	betweenness centrality
1	Zigong City	Zigong City	Panzhihua	25	Zunyi City	Zunyi City	Lijiang
2	Yibin	Yibin	Dali Prefecture	26	Anshun City	Bazhong City	Yuxi City
3	Neijiang City	Panzhihua	Qujing City	27	Liupanshui	Guangyuan City	Anshun City
4	Luzhou	Qujing City	Zigong City	28	Tongren City	Tongren City	Chongqing
5	Ziyang City	Luzhou	Baoshan	29	Qiannan Prefecture	Chuxiong Prefecture	Suining City
6	Chengdu	Neijiang City	Yibin	30	Qianxinan Prefecture	Dali Prefecture	Zunyi City
7	Deyang City	Leshan	Lincang	31	Aba Prefecture	Kunming	Nanchong
8	Qujing City	Zhaotong	Luzhou	32	Chuxiong Prefecture	Yuxi City	Guang’an
9	Suining City	Liangshan Prefecture	Guiyang City	33	Dali Prefecture	Lijiang	Aba Prefecture
10	Nanchong	Ziyang City	Pu’er City	34	Kunming	Qiannan Prefecture	Dazhou
11	Guang’an	Guiyang City	Chengdu	35	Yuxi City	Qianxinan Prefecture	Meishan City
12	Mianyang City	Liupanshui	Neijiang City	36	Baoshan	Nujiang Prefecture	Kunming
13	Dazhou	Chengdu	Liangshan Prefecture	37	Ganzi Prefecture	Honghe Prefecture	Bazhong City
14	Guiyang City	Bijie City	Leshan	38	Lijiang	Wenshan Prefecture	Qianxinan Prefecture
15	Leshan	Deyang City	Chuxiong Prefecture	39	Qiandongnan Prefecture	Aba Prefecture	Ganzi Prefecture
16	Panzhihua	Suining City	Ziyang City	40	Honghe Prefecture	Ganzi Prefecture	Dehong Prefecture
17	Bazhong City	Nanchong	Tongren City	41	Lincang	Qiandongnan Prefecture	Guangyuan City
18	Meishan City	Guang’an	Ya’an city	42	Nujiang Prefecture	Baoshan	Honghe Prefecture
19	Chongqing	Mianyang City	Liupanshui	43	Pu’er City	Lincang	Nujiang Prefecture
20	Bijie City	Chongqing	Deyang City	44	Dehong Prefecture	Dehong Prefecture	Qiandongnan Prefecture
21	Guangyuan City	Anshun City	Zhaotong	45	Wenshan Prefecture	Pu’er City	Wenshan Prefecture
22	Ya’an city	Dazhou	Bijie City	46	Xishuangbanna Prefecture	Xishuangbanna Prefecture	Xishuangbanna Prefecture
23	Zhaotong	Meishan City	Qiannan Prefecture	47	Diqing Prefecture	Diqing Prefecture	Diqing Prefecture
24	Liangshan Prefecture	Ya’an city	Mianyang City	Average	20.999	3.489	28.183

Point-degree centrality can be used to measure the radiation ability or agglomeration ability of node cities in the network. Table 3 shows that the average point degree centrality of cities in the upper reaches of the Yangtze River is 20.999, and the top ten cities are Zigong City, Yibin City, Neijiang City, Luzhou City, Ziyang City, Chengdu City, Deyang City, Qujing City, Suining City, and Nanchong City, indicating that these cities are in the center of the entire spatial correlation network and have more connections with other nodes. These cities (autonomous prefectures) play a key role in the formation and stable development of the overall network. Most of these cities are located in Sichuan. Compared to other areas, the basin area has faster economic development, superior natural conditions, and more convenient transportation conditions. It is also a densely populated area in Sichuan Province, and it is an important consumption and production area for agricultural products. However, the degree of centrality of cities (autonomous prefectures) in Yunnan is relatively low. Compared with the areas at the center of the network, the economic development speed is relatively slow, and the ecological efficiency of cultivated land utilization is not high.

Proximity centrality reflects the connectivity of the network. The average value of the centrality of the upper reaches of the Yangtze River is 3.489, and the top ten regions include Zigong City, Yibin City, Panzhihua City, Qujing City, Luzhou City, Neijiang City, Leshan City, Zhaotong City, Liangshan Prefecture, and Ziyang City, indicating that these regions play a role as "bridges" in the spatial network of cultivated land utilization ecological efficiency and have a strong control ability in the network structure. Moreover, these areas are mostly located in the center of the research area, and the correlations in the entire network are mainly completed through these areas, and thus they can better regulate the cross-regional flow of agricultural resource elements such as production technology, labor force, and capital and can influence other areas. Regions play a regulating and restricting role.

Betweenness centrality can reflect whether a node in the network is prone to spatial association with other nodes. The average betweenness centrality of the upper reaches of the Yangtze River is 28.183, and the top ten regions are Panzhihua City, Dali Prefecture, Qujing City, Zigong City, Baoshan City, Yibin City, Lincang City, Luzhou City, Guiyang City, and Pu’er City. This reflects that these node cities (autonomous prefectures) are relatively close to other nodes in the spatial correlation network and can quickly establish association relationships with other nodes, thereby driving the improvement of the ecological efficiency of cultivated land utilization in other areas.

#### Network cohesion subgroup analysis

**Fig 5:**
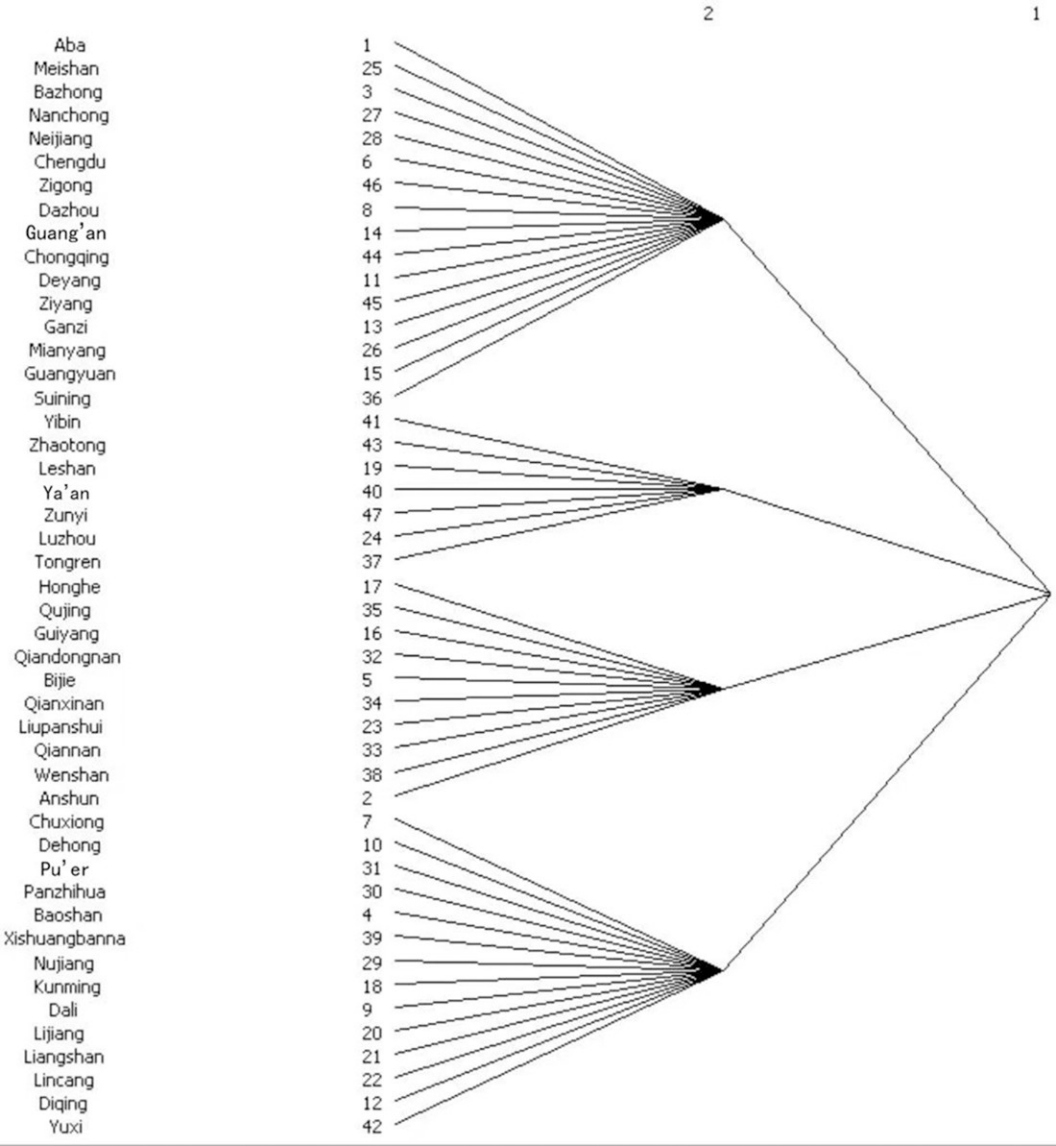
Cohesive subgroups of the ecological efficiency network of cultivated land utilization in the upper reaches of the Yangtze River in 2020.

Through the function of CONCOR (Iterative Correlation Convergence Method) in Ucinet 6 software, cohesive subgroup analysis of the ecological efficiency network of cultivated land utilization in the upper reaches of the Yangtze River was carried out, and the maximum segmentation depth was set to 2. As shown in Fig 5, the cities (autonomous prefectures) in the upper reaches of the Yangtze River are divided into four cohesive subgroups on the second-level branch, and there are no isolated cities. Among these subgroups, subgroup 1 has the most cities, including 16 cities in total, and most of them are located in Sichuan Province; Subgroup 2 includes 7 cities; Subgroup 3 includes 10 cities, mostly located in Guizhou Province; and Subgroup 3 includes 14 cities, mostly in Yunnan Province.

The subgroup density and its image matrix are shown in Table 4, among which subgroup 1 has the highest density of 0.708, indicating that the cities (autonomous prefectures) within subgroup 1 communicate frequently and are closely connected. The density of subgroup 4 is the smallest, which is 0.176, indicating that there is less communication among members within the subgroup, and the spatial correlation within the subgroup is not too strong. Among them, subgroup 1 not only has a large number of internal relations but also receives spillover effects from Subgroup 2 and has an impact on Subgroup 2. Subgroup 2 receives the spillover effects of Subgroup 1 and Subgroup 3 at the same time, and Subgroup 3 has a spatial spillover relationship with Subgroup 2 and Subgroup 4.

**Table 4:** The cohesive subgroup density matrix of cultivated land utilization ecological efficiency network.

Density matrix					Like matrix			
Subgroup	1	2	3	4	1	2	3	4
1	0.708	0.295	0.006	0.004	1	1	0	0
2	0.357	0.548	0.157	0.071	1	1	0	0
3	0.031	0.214	0.456	0.029	0	1	1	1
4	0.009	0.031	0.050	0.176	0	0	0	0

**Fig 6:**
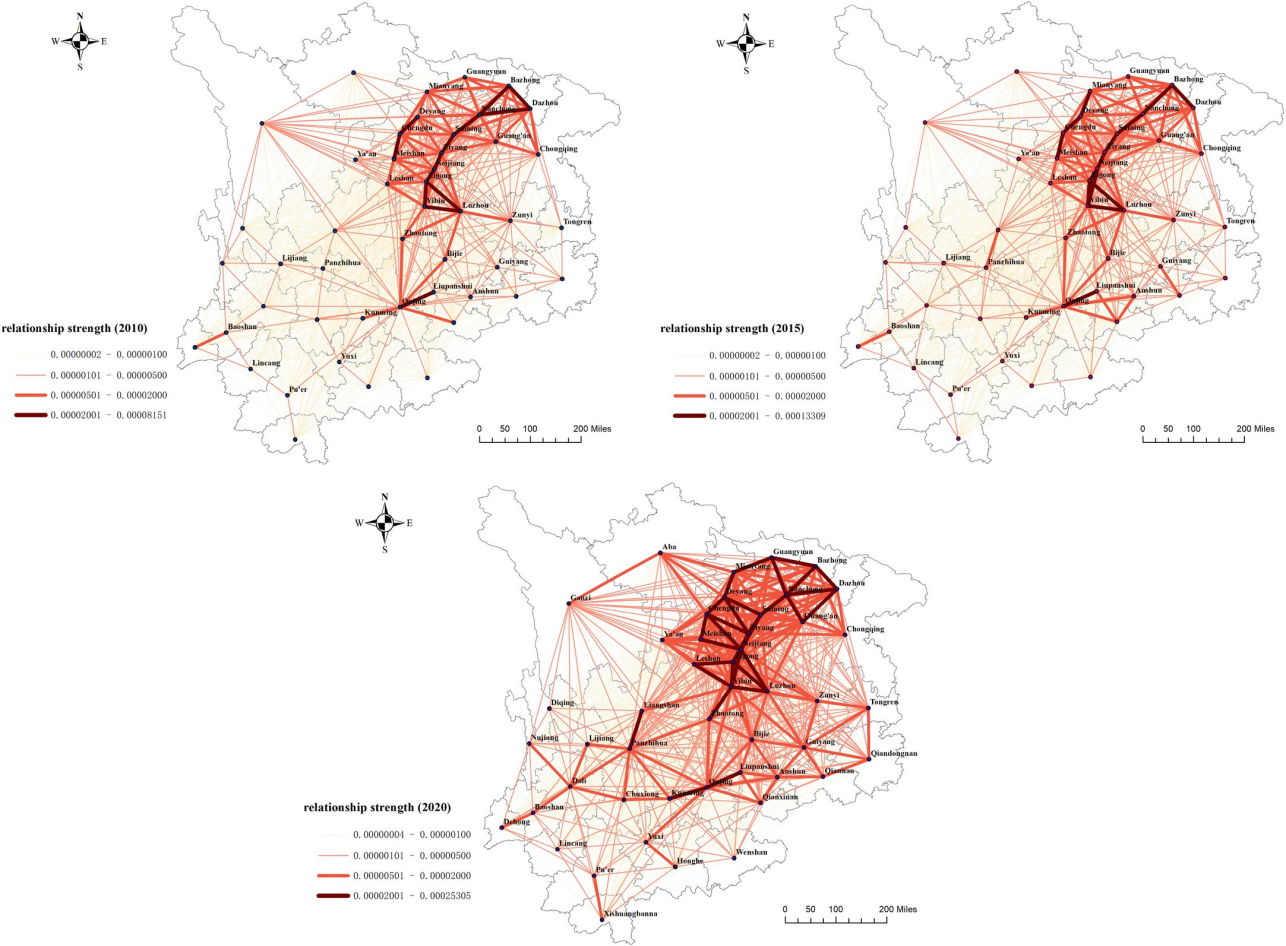
Spatial distribution of cohesive subgroups in the upper reaches of the Yangtze River. The map was prepared with ArcGIS10.1 (https://resources.arcgis.com/en/help/). The administrative boundary was republished from [34] under a CC BY license, with permission from Resource and Environment Science and Data Center (https://www.resdc.cn/), original copyright 2023.

Fig 6 shows the spatial distribution of cohesive subgroups of the second-level branches. The spatial correlation of the ecological efficiency of cultivated land utilization in the upper reaches of the Yangtze River has broken through the provincial boundaries, it shows the aggregation and coupling of agricultural elements in space. Cohesive subgroup 1 is located in the northern part of the study area, and its point-degree centrality, closeness centrality, and betweenness centrality values in Zigong City, Chengdu City, and Neijiang City are all higher. It shows that these cities have a great influence on the use of cultivated land in other cities and have a strong intermediary ability. Most members of the subgroup are located in the Chengdu-Chongqing region. The conditions of cultivated land are superior, the economy develops rapidly, and the transportation network is completed. It can gather a large number of agricultural resources and radiate the entire upper reaches of the Yangtze River. In subgroup 3, most of the members of the subgroup are located in Guizhou Province, among which the comprehensive ranking of betweenness centrality, proximity centrality, and point degree centrality in Guiyang City ranks first, and the centrality index is the best in the subgroup. Guiyang, located in the middle of Guizhou Province, is one of the important central cities in Southwest China. Due to relatively good economic conditions in Guiyang, a high level of agricultural technology, and relatively high economic and comprehensive benefits, it radiates to other regions. In subgroup 4, most of the members are located in Yunnan Province, among which Panzhihua City’s betweenness centrality, proximity centrality, and point degree centrality are ranked first, and Panzhihua City is located in the central area of the subgroup. Since the "13th Five-Year Plan", Panzhihua City has made full use of the unique advantages of light and heat climate resources in the dry and hot valley of the Jinsha River, vigorously implemented the rural revitalization strategy, and promoted the development of characteristic and efficient agriculture. It is an important fruit production base in Sichuan Province and has been included in the national modern agricultural area. The total agricultural output value of the area has increased, and the efficiency of cultivated land utilization has also increased, thereby radiating to the surrounding areas.

### Influencing factors analysis

To avoid measurement error caused by multiple linearities, this paper uses the QAP method commonly used in social network analysis to explore the influencing factors of the spatial correlation of cultivated land utilization ecological efficiency in the upper reaches of the Yangtze River in 2020 and selects 5000 permutations for random row and column replacement. Since the spatial correlation of Ecological efficiency of cultivated land utilization is closely related to the diffusion of agricultural elements in different regions, this paper refers to previous research results and believes that the factors affecting the spatial correlation of ecological benefits of cultivated land utilization are shown in Table 5.

**Table 5:** Influencing factors and variable description of the spatial network of cultivated land utilization ecological efficiency.

Serial number	Influencing factors	Variable description
X1	Differences in the level of agricultural economic development	The absolute difference in the ratio of regional agricultural output value to GDP
X2	The difference in the urbanization rate	Absolute Differences in Regional Urbanization Rates
X3	Rural Per Capita Disposable Income Difference	Absolute Differences in Regional Rural Per Capita Disposable Income
X4	Differences in Fertilizer Intensity	The absolute difference in the ratio of fertilizer application intensity to crop sown area in different regions
X5	Differences in Agricultural Mechanization Intensity	The absolute difference in the ratio of the total power of agricultural machinery to the sown area of crops in different regions
X6	Regional Population Differences	The absolute differences in the number of permanent residents in different regions
*S*	spatial adjacency	If the geographical boundary is adjacent, it is recorded as 1, and if it is not adjacent, it is recorded as 0

The indicators are explained as follows: (1) Differences in the level of agricultural economic development (X1). In this paper, the ratio of total agricultural output value to GDP in each region is selected to represent the level of agricultural economic development. Usually, the smaller the difference in the level of agricultural economic development between regions, the more conducive it is to the formation of spatial networks. (2) Difference in urbanization rate (X2). Usually, the urbanization rate will inhibit the ecological efficiency of agricultural utilization in the region and surrounding areas. The increase in the urbanization rate will lead to the non-agriculturalization of cultivated land, the outflow of the rural population to the city, and the "siphon effect". Other issues affect the formation of the network. Scholars have found that urbanization can affect the ecological efficiency of cultivated land utilization by affecting the input-output combination of cultivated land utilization [39]. (3) Difference in rural per capita disposable income (X3). Generally, the level of people’s income affects the input of farmers’ capital, technology, labor, and other factors and can also increase farmers’ enthusiasm for agricultural production. At the same time, the flow of economic factors between regions will also promote the improvement of the economic level of surrounding areas, thereby improving agricultural production conditions. (4) Differences in fertilizer application intensity (X4). This paper selects the ratio of the amount of chemical fertilizer application to the sown area of crops in each region for representation. Generally, the increase in the intensity of chemical fertilizer use can increase the yield in a short period, but it will increase soil nonpoint source pollution. Soil quality in the region will affect the overall ecological efficiency of cultivated land utilization. (5) Differences in the intensity of agricultural mechanization (X5). In this paper, the total power of agricultural machinery and the sown area of crops in each region are selected to represent the intensity of agricultural mechanization. The intensity of agricultural mechanization can reflect the level of agricultural technology [40]. Usually, the higher the level of agricultural technology, the better it is to save time and improve the saving of agricultural labor costs. However, if the gap between regions is too large, such a gap will not be conducive to the spillover of agricultural technology, will hinder the spread of advanced agricultural production technology, and thus will not be conducive to the formation of spatial networks. (6) Regional population differences (X6). This paper selects the number of permanent residents in each region to represent the population of the region [41]. Usually, the number of people affects the number of agricultural laborers in a region. The increase in the number of workers will also promote the transformation of agricultural production methods and is also conducive to the refined management of cultivated land. (7) Spatial adjacency (*S*). According to the first law of geography, geographical things are related to each other in terms of spatial distribution. Generally, the closer the distance, the stronger the correlation, and this can promote the exchange of agricultural elements.

According to the QAP regression analysis results, the R^2^ is 0.592, and the adjustment R^2^ is 0.586, indicating that the explanatory power of the selected variable on the formation of the spatial correlation network of ecological efficiency of cultivated use in the upper reaches of the Yangtze River is about 58.6%, explain that the model has strong explanatory power [1].

From the results of the QAP correlation analysis (Table 6), the correlation coefficient of the agricultural economic development level difference matrix is 0.0074, the correlation coefficient of the rural per capita disposable income difference matrix is 0.11, and the correlation coefficient of the spatial adjacency matrix is 0.51, all of which have passed the significance test. It shows that the differences in the level of agricultural economic development(x1), the rural per capita disposable income difference (X3), the differences in agricultural mechanization intensity(x5), the regional population differences(x6), and the spatial adjacency(*S*) have a relatively significant correlation with the spatial network of cultivated land utilization Ecological efficiency. The P values of other independent variables have not passed the significance test, indicating that the correlation with the spatial network of cultivated land utilization Ecological efficiency was not strong.

**Table 6:** QAP analysis of network factors of cultivated land utilization ecological efficiency.

Independent variable	QAP correlation analysis		QAP regression analysis	
	Correlation coefficient	P value	Regression coefficients	P value
X1	-0.074	0.061*	-0.005878	0.066*
X2	-0.021	0.360	-0.001589	0.260
X3	-0.110	0.500	-0.000012	0.016*
X4	-0.0045	0.520	-0.003164	0.145
X5	-0.0085	0.0084**	-0.000252	0.098*
X6	0.049	0.026*	0.000201	0.005***
*S*	0.510	0.0002***	0.621900	0.000***

Note:

***, **, * represent P<0.01, P<0.05, P<0.1, respectively; if P<0.1, it is significant.

Among the regression coefficients, the differences in the level of agricultural economic development(x1) are significantly negative, indicating that the closer the level of agricultural economic development between regions is, the more conducive it is to strengthen the spatial relationship of ecological efficiency of cultivated land utilization in the upper reaches of the Yangtze River. The higher the level of agricultural economic development, and the smaller the gap, the more frequent the flow of production factors such as production technology and equipment between regions, promoting the improvement of production efficiency in the entire region and thus promoting the formation of social networks. The rural per capita disposable income difference (X3) matrix is significantly negative, indicating that the closer the disposable income levels of rural residents between regions are, the more conducive it is to promote the formation of a spatial network of ecological efficiency of cultivated land use. This is because the flow of agricultural factors such as resources, technology, and labor flows more frequently between regions with similar economic levels, making it easier to form a spatial correlation network. The differences in the agricultural mechanization Intensity(x5) matrix are significantly negative, indicating that the closer the agricultural mechanization intensity is, the more conducive it is to strengthen the spatial network relationship of the ecological efficiency of cultivated land utilization in the upper reaches of the Yangtze River. The improvement of the level of agricultural mechanization is conducive to improving labor production efficiency and saving labor costs, and the popularity of cross-regional operations of agricultural machinery has promoted the exchange of agricultural machinery elements, thereby promoting the formation of the ecological efficiency network of cultivated land utilization. The regional population differences(x6) matrix being significantly positive indicates that the population gap in different regions can promote the formation of cultivated land utilization eco-efficiency networks in the upper reaches of the Yangtze River. The difference in population status is directly reflected in the difference in per capita cultivated land resources, forming the reality of labor flow from areas with less per capita cultivated land to areas with more per capita cultivated land. While reducing the ecological environment pressure in areas with less per capita cultivated land, it will also significantly improve the efficiency of cultivated land utilization, thereby promoting the flow of factors and network formation between regions. The spatial adjacency(*S*) matrix is significantly positive, indicating that the spatial network of ecological efficiency of cultivated land utilization in the upper reaches of the Yangtze River has a significant spatial correlation, that is, the closer the distance between regions is, the conducive to the flow of agricultural resource elements, forming a significant social network of Ecological efficiency of cultivated land utilization.

## Discussion

Based on the above results and analysis, several interesting phenomena were highlighted. First, from the perspective of the evolution of cultivated land use ecological efficiency, the cultivated land use efficiency in the upper reaches of the Yangtze River is generally not high and is lower than the cultivated land use efficiency in the middle reaches of the Yangtze River [42], but it shows an increasing trend, which is consistent with China’s cultivated land use efficiency in recent years. The dynamic evolution characteristics are relatively consistent with [43]. Sichuan and other places are significantly higher than Guizhou and other places, showing a relatively obvious spatial differentiation pattern. All localities should formulate relevant policies to improve the ecological efficiency of cultivated land use in an overall and balanced manner to promote the coordinated development of green and efficient agriculture in the upper reaches of the Yangtze River [44], Sichuan Province has a high level of ecological efficiency in cultivated land utilization. When formulating policies, it should give full play to its advantages, guide the green development of agriculture, promote the development of modern agriculture, and improve ecological service functions. The terrain of Yunnan Province changes with fluctuations, so it should make full use of its light and heat conditions and resource advantages, actively guide the development of regional characteristic agriculture, and expand agricultural production and agricultural product processing industries with comparative advantages; Guizhou Province is limited by the influence of complex karst landforms and arable land conditions When using cultivated land, we should pay more attention to the scientific nature of cultivated land, avoid over-use of cultivated land, and strengthen the rest and rotation of cultivated land, which can not only protect the fertility of cultivated land but also improve the efficiency of cultivated land use [45].

Secondly, although the inter-provincial ecological efficiency of cultivated land use in the upper reaches of the Yangtze River has formed an obvious spatial correlation network and cohesive subgroups, the tightness and stability of the network are not enough, indicating that the inter-provincial spatial spillover effect of cultivated land use efficiency is not enough. Significantly [46], the formation of spatial networks is not only affected by spatial adjacent relationships but also by various factors such as the level of economic development[47]. Promoting the improvement of cultivated land utilization efficiency should comply with the laws of spatial correlation [48]. On the one hand, we should pay attention to the impact of spatial distance, improve the transportation network in the upper reaches of the Yangtze River, and create convenient conditions for the flow of agricultural production factors [44]. For areas with good cultivated land conditions and abundant labor resources, encourage the transfer of their labor force to higher value-added industries, affecting the opportunity cost of cultivated land utilization, thereby improving the ecological efficiency of cultivated land use [49]. On the other hand, government departments should strengthen the publicity and promotion of the concept of green cultivated planting, carry out agricultural technology training and exchange activities, promote the use of green pesticides and organic fertilizers, and reduce cultivated source pollution [7].

At the same time, there are still some limitations in the research that need to be further explored and deepened. For example, due to the lack of agricultural data in Guizhou and Yunnan provinces, indicators such as pesticides and land irrigation areas were not included in the system. In addition, this article only analyzes the network structure and influencing factors of cultivated land utilization ecological efficiency in 2020, and further research on the changes in its impact is still needed. In future research, the evaluation index system for cultivated utilization efficiency can be further improved, fully considering the value of the cultivated ecosystem, and incorporating more ecological environment indicators into unexpected outputs to enhance the sustainability of cultivated utilization. In terms of influencing factors, attention should also be paid to factors such as improving farming systems, investing in new agricultural machinery, and adjusting agricultural structures; In terms of research areas, research should be conducted on specific areas (such as grain production areas, ecological protection areas, etc.) and special areas (such as mountainous areas, river basins, river valleys, etc.), providing reference for local governments to formulate targeted policies to improve cultivated land utilization efficiency.

## Conclusion

This paper uses the super-efficiency SBM model to calculate the ecological efficiency of cultivated land utilization in 47 cities (autonomous prefectures) in the upper reaches of the Yangtze River from 2010 to 2020. Based on this, it analyzes the spatial correlation of the ecological efficiency of cultivated land utilization in the upper reaches of the Yangtze River, calculates the overall network density, network centrality, and other indicators, conducts cohesive subgroup analysis on the ecological efficiency network of cultivated land utilization in the upper reaches of the Yangtze River, and finally uses the QAP model to discuss the influencing factors of the spatial correlation network of ecological efficiency in cultivated land utilization. The results show the following:

1. The ecological efficiency of cultivated land utilization in the upper reaches of the Yangtze River is generally low, but it shows a steady overall growth trend. The overall average value rose from 0.396 in 2010 to 0.623 in 2020. The average value in Sichuan Province is the highest, reaching medium efficiency. The average value of Yunnan Province in 2010 and 2020 is in the low-efficiency zone. The ecological efficiency of cultivated land utilization in Guizhou Province has steadily increased year by year, from 0.311 in 2010 to 0.603 in 2020, from low efficiency to medium efficiency.
2. The ecological efficiency of cultivated land utilization in the upper reaches of the Yangtze River breaks through the spillover effect of adjacent geographical space and presents a certain spatial network structure. Cities such as Yibin, Chengdu, and Zigong are at the center of the spatial correlation network, and most of the cities in the center of the spatial network are located in the Sichuan Basin, while the cities (autonomous prefectures) in Yunnan and Guizhou provinces are mostly at the edge of the association network and they communicate with each other loosely. From the results of cohesive subgroup analysis, it can be seen that the spatial correlation network of cultivated land utilization ecological efficiency in the upper reaches of the Yangtze River can be divided into four subgroups, and the cities within the subgroups are frequently connected, among which subgroup 1 is the most closely connected, there are also exchanges among them. The spatial division of provincial boundaries has not affected the flow of agricultural elements in the upper reaches of the Yangtze River and the spatial correlation of cultivated land utilization.
3. In terms of influencing factors, the differences in the level of agricultural economic development, the differences in agricultural mechanization intensity, the regional population differences, the spatial adjacency have a relatively significant correlation with the ecological efficiency spatial network of cultivated land utilization in the upper reaches of the Yangtze River. The difference in the level of agricultural economic development and the difference in the intensity of mechanization intensity are negatively correlated, indicating that the smaller the difference in these conditions in different regions, the more conducive to the flow of factors and the formation of social networks. The regional population difference and the spatial adjacency are positively correlated, indicating that population difference and spatial distance will further accelerate the flow of labor and other factors, and promote the formation of social network.

## Supporting information

S1 File (XLSX)
